# Supplementary material for: Continuum beliefs in the stigma process regarding persons with schizophrenia and depression: results of path analyses
Source: PeerJ. 2016 Sep 27;4:e2360. doi: 10.7717/peerj.2360 (PMC5045891; doi:10.7717/peerj.2360)
Supplement: Supplemental Information 3 [file peerj-04-2360-s003.docx]

ANNEX

Additional Correlation Analyses

**Table A1** Correlations between continuum belief, stereotypes, emotional reactions and desire for social distance (Schizophrenia)

|  | Continuum Belief | Unpredictable | Dangerous | Anger | Fear | Prosocial |
| --- | --- | --- | --- | --- | --- | --- |
| Unpredictable | -0.015 |  |  |  |  |  |
| Dangerous | -0.004 | 0.464^**^ |  |  |  |  |
| Anger | 0.030 | 0.206^**^ | 0.206^**^ |  |  |  |
| Fear | -0.060^*^ | 0.301^*^ | 0.306^**^ | 0.387^**^ |  |  |
| Prosocial | 0.073^**^ | -0.004 | -0.063^*^ | -0.077^**^ | -0.043 |  |
| Desire for social distance | -0.090^**^ | 0.378^**^ | 0.378^**^ | 0.205^**^ | 0.285^**^ | -0.242^**^ |

Pearson’s r; ^*^p<0.05 ^**^p<0.01

Table A1 displays bivariate associations between (dis-) agreement in continuity of symptoms and stigma components (stereotypes, emotional reactions, desire for social distance) regarding schizophrenia. The continuum belief item is positively related to prosocial emotional reactions. Furthermore, stronger agreement with continuity of symptoms is associated with reduced desire for social distance and feelings of fear. There are no statistically significant correlations between continuum belief and stereotypes.

**Table A2** Correlations between continuum belief, stereotypes, emotional reactions and desire for social distance (Depression)

|  | Continuum Belief | Unpredictable | Dangerous | Anger | Fear | Prosocial |
| --- | --- | --- | --- | --- | --- | --- |
| Unpredictable | 0.027 |  |  |  |  |  |
| Dangerous | 0.017 | 0.372^**^ |  |  |  |  |
| Anger | 0.019 | 0.193^**^ | 0.271^**^ |  |  |  |
| Fear | 0.062^*^ | 0.275^**^ | 0.263^**^ | 0.436^**^ |  |  |
| Prosocial | 0.118^**^ | 0.037 | 0.013 | -0.146^**^ | 0.015 |  |
| Desire for social distance | -0.127^**^ | 0.226^**^ | 0.277^**^ | 0.294^**^ | 0.275^**^ | -0.215^**^ |

Pearson’s r; ^*^p<0.05 ^**^p<0.01

Regarding depression, results of the bivariate correlation analyses are displayed in table A2. Similar to schizophrenia, there are no statistically significant associations between (dis-) agreement with continuity of symptoms and stereotypes. However, stronger agreement with the continuum belief item is associated with less desire for social distance and greater prosocial reactions. Interestingly, the relation between belief in continuity of symptoms and feelings of fear is positive.
