# Supplementary material for: Continuum beliefs in the stigma process regarding persons with schizophrenia and depression: results of path analyses
Source: PeerJ. 2016 Sep 27;4:e2360. doi: 10.7717/peerj.2360 (PMC5045891; doi:10.7717/peerj.2360)
Supplement: Supplemental Information 4 [file peerj-04-2360-s004.docx]

**Table A1** Correlations between continuum belief, stereotypes, emotional reactions and desire for social distance (Schizophrenia)

|  | Continuum Belief | Unpredictable | Dangerous | Anger | Fear | Prosocial |
| --- | --- | --- | --- | --- | --- | --- |
| Unpredictable | -0.015 |  |  |  |  |  |
| Dangerous | -0.004 | 0.464^**^ |  |  |  |  |
| Anger | 0.030 | 0.206^**^ | 0.206^**^ |  |  |  |
| Fear | -0.060^*^ | 0.301^*^ | 0.306^**^ | 0.387^**^ |  |  |
| Prosocial | 0.073^**^ | -0.004 | -0.063^*^ | -0.077^**^ | -0.043 |  |
| Desire for social distance | -0.090^**^ | 0.378^**^ | 0.378^**^ | 0.205^**^ | 0.285^**^ | -0.242^**^ |

^*^p<0.05 ^**^p<0.01
