# Supplementary material for: Continuum beliefs in the stigma process regarding persons with schizophrenia and depression: results of path analyses
Source: PeerJ. 2016 Sep 27;4:e2360. doi: 10.7717/peerj.2360 (PMC5045891; doi:10.7717/peerj.2360)
Supplement: Supplemental Information 5 [file peerj-04-2360-s005.docx]

**Table A2** Correlations between continuum belief, stereotypes, emotional reactions and desire for social distance (Depression)

|  | Continuum Belief | Unpredictable | Dangerous | Anger | Fear | Prosocial |
| --- | --- | --- | --- | --- | --- | --- |
| Unpredictable | 0.027 |  |  |  |  |  |
| Dangerous | 0.017 | 0.372^**^ |  |  |  |  |
| Anger | 0.019 | 0.193^**^ | 0.271^**^ |  |  |  |
| Fear | 0.062^*^ | 0.275^**^ | 0.263^**^ | 0.436^**^ |  |  |
| Prosocial | 0.118^**^ | 0.037 | 0.013 | -0.146^**^ | 0.015 |  |
| Desire for social distance | -0.127^**^ | 0.226^**^ | 0.277^**^ | 0.294^**^ | 0.275^**^ | -0.215^**^ |

^*^p<0.05 ^**^p<0.01
